# Are SGLT2 polymorphisms linked to diabetes mellitus and cardiovascular disease? Prospective study and meta-analysis

**DOI:** 10.1042/BSR20190299

**Published:** 2019-08-07

**Authors:** Heinz Drexel, Andreas Leiherer, Christoph H. Saely, Eva Maria Brandtner, Kathrin Geiger, Alexander Vonbank, Peter Fraunberger, Axel Muendlein

**Affiliations:** 1 Vorarlberg Institute for Vascular Investigation and Treatment (VIVIT), Feldkirch, Austria; 2 Division of Angiology, Swiss Cardiovascular Center, University Hospital of Berne, Berne, Switzerland; 3 Private University of the Principality of Liechtenstein, Triesen, Liechtenstein; 4 Drexel University College of Medicine, Philadelphia, PA, U.S.A.; 5 Medical Central Laboratories, Feldkirch, Austria; 6 Department of Medicine and Cardiology, Academic Teaching Hospital Feldkirch, Feldkirch, Austria

**Keywords:** cardiovascular disease, SGLT2, single nucleotide polymorphisms, type 2 diabetes

## Abstract

Inhibition of the sodium glucose co-transporter 2 (SGLT2) reduces cardiovascular morbidity, and mortality in patients with type 2 diabetes mellitus (T2DM) with atherosclerotic, cardiovascular disease. So far, a link between common genetic variations of the SGLT2 encoding gene SLC5A2 and glucose homeostasis as well as cardiovascular disease has not been established. The present study, therefore, aimed to investigate SLC5A2 single nucleotide polymorphisms (SNPs) in relation to type 2 diabetes and coronary artery disease (CAD) and prospectively the incidence of cardiovascular events. We genotyped the SLC5A2 tagging SNPs rs9934336, rs3813008, and rs3116150 in a total of 1684 high risk cardiovascular patients undergoing coronary angiography, including 400 patients with T2DM. Additionally, we performed a meta-analysis combining results from the present study and the literature. Variant rs9934336 was significantly associated with decreased HbA1c (*P* = 0.023). Further, rs9934336 was significantly inversely associated with the presence of T2DM in univariate (OR = 0.82 [0.68–0.99]; *P* = 0.037) as well as in multivariate analysis (OR = 0.79 [0.65–0.97]; *P* = 0.023). The association between rs9934336 and T2DM was confirmed in a meta-analysis including results from two previous observations which by themselves had failed to show a significant association of the polymorphism with T2DM (OR = 0.86 [0.78–0.95]; *P* = 0.004). Polymorphisms rs3813008 and rs3116150 were associated neither with glycemic parameters nor with T2DM. None of the SNPs tested was significantly associated with the baseline presence of CAD or the incidence of cardiovascular events. We conclude that genetic variation within the SLC5A2 gene locus is significantly related to the manifestation of T2DM.

## Introduction

To preserve energy, glomerular filtered glucose is physiologically reabsorbed in the proximal tubule by the high-capacity sodium glucose co-transporter 2 (SGLT2) and the high-affinity SGLT1 transporter. This reabsorption frees the filtrate almost totally from glucose, cotransports sodium and is increased with a higher filtered glucose load like in hyperglycemia and therefore in diabetes [1].

The SGLT2 molecules – formerly mainly of nephrological interest [2] – gained the attention of diabetologists when specific inhibitors became available [3]. Interest also was shared by cardiologists when the results of the Empagliflozin, Cardiovascular Outcomes, and Mortality in Type 2 Diabetes (EMPA-REG-OUTCOME) trial revealed that the SGLT2 inhibitor empagliflozin, compared with standard antidiabetic treatment, reduced cardiovascular mortality and morbidity in type 2 diabetic patients with established cardiovascular disease [4,5]. The Canagliflozin Cardiovascular Assessment Study essentially confirmed these findings with the SGLT2 inhibitor canagliflozin [6]. So far, the underlying mechanisms behind the cardiovascular protection observed in clinical trials of SGLT2 inhibitors remain incompletely understood and there is a great scientific interest in potential pathways linking SGLT2 to diabetes and cardiovascular disease. Genetic analyses in many other fields have substantially helped to understand mechanisms and to reveal causal relationships [7–9].

SGLT2 is encoded by the SLC5A2 gene on chromosome 16 and is expressed almost exclusively in the proximal renal tubule. It has been hypothesized – but never proven – that a loss of function mutation in SLC5A2 could protect from T2DM by elimination of glucose in an insulin-independent mode via glucosuria. Testing common variants in the SLC5A2 gene in large patient cohorts could shed some new light on the association between SLC5A2 and the pathogenesis of T2DM. However, until now, the association between common genetic variations in SLC5A2 and glucose homeostasis or T2DM has hardly been investigated and the few published reports provided inconclusive results [10,11]. Moreover, the association between SLC5A2 variants and coronary atherosclerosis as well as with the risk of future cardiovascular events is unclear.

We therefore analyzed three tagging SNPs of the SLC5A2 gene in a large cohort of patients characterized by coronary angiography, including a high proportion of T2DM. We explored whether SLC5A2 SNPs were associated with (i) fasting glucose, the post-challenge glucose increase in oral glucose tolerance tests and HbA1c; (ii) the prevalence of T2DM, and (iii) baseline coronary artery disease (CAD) as well as incidence of cardiovascular events during follow-up. In addition, we performed a meta-analysis including data from earlier studies to further elucidate the impact of genetic variants of the SLC5A2 gene on the presence of diabetes.

## Materials and methods

### Study subjects

We enrolled a total of 1684 consecutive Caucasian patients, who were referred to elective coronary angiography for the evaluation of established or suspected stable CAD at the academic teaching hospital Feldkirch, a tertiary care center in Western Austria, as described previously [12,13].

Hypertension was defined according to the Seventh Report of the *Joint National Committee on Prevention, Detection, Evaluation, and Treatment of High Blood Pressure* [14], and T2DM was diagnosed according to *World Health Organization* criteria [15]. Height and weight were recorded, and body mass index (BMI) was calculated as body weight (kg)/height (m^2^). The metabolic syndrome was diagnosed according to *National Cholesterol Education Program* ATP-III criteria [16]. Coronary angiography was performed with the Judkins technique [17]. Stenosis of the coronary arteries was considered significant when lumen narrowing was ≥50% [18]. The extent of coronary atherosclerosis was defined as the number of significant coronary stenoses in a given patient, as described previously [19,20]. During a mean (±SD) follow-up period of 7.6 ± 2.7 years, fatal as well as non-fatal cardiovascular events were recorded. Cardiovascular events were defined as a composite of coronary death (fatal myocardial infarction, sudden cardiac death, mortality from congestive heart failure due to CAD), fatal ischemic stroke, non-fatal myocardial infarction, non-fatal ischemic stroke and need for aorto-coronary bypass surgery, percutaneous transluminal coronary angioplasty, or revascularization in the carotid or peripheral arterial beds. This primary study endpoint has been *a priori* chosen in analogy to a large cardiovascular outcome trial, the Heart Protection Study [21].

The Ethics Committee of the Medical University of Innsbruck approved the present study; all participants gave written informed consent and all clinical investigations have been conducted according to the principles expressed in the Declaration of Helsinki.

### Laboratory analyses

Venous blood samples were collected after an overnight fast of 12 h before angiography was performed. Serum triglycerides and total cholesterol were determined on a Cobas 6000 or 8000 (Roche, Basel, Switzerland).

Levels of fasting plasma glucose were measured enzymatically from venous fluoride plasma samples with the hexokinase method (Roche Basel, Switzerland) on a Hitachi 717 or 911 (Mountain View, CA, U.S.A.). Glycosylated hemoglobin was determined as HbA1c by high-performance liquid chromatography on a Menarini-Arkray KDK HA 8140 (Kyoto, Japan).

Oral glucose tolerance tests were performed after an oral 75g glucose challenge. Serum insulin was measured by an enzyme immunoassay on an AIA 1200 (Tosoh, Foster City, CA, U.S.A.). Further, beta-cell function and insulin resistance were estimated from fasting plasma glucose and serum insulin using homeostasis model assessment [22].

### Genotyping

Three SLC5A2 SNPs were selected due to their given roles as tagging SNPs. According to data of the 1000 Genomes Project, pilot 1 [23], or HapMap SNP database, release 27 [24], these SNPs cover all variants with a minor allele frequency ≥ 0.05 and pairwise *r*^2^ ≥ 0.8 within the SLC5A2 gene including 2 kb of the 5′ flanking region and 1 kb of the 3′ flanking region. Genotyping of these variants was carried out by the 5′ nuclease assay using TaqMan^®^ MGB probes on a LightCycler^®^ 480 Real-Time PCR System (F. Hoffmann-La Roche Ltd, Basel, Switzerland). TaqMan^®^ MGB probes together with corresponding PCR primers were provided by the Assay-on-demand™ service (Thermo Fisher Scientific, Waltham, MA, U.S.A.). Genotypes were automatically determined by LightCycler^®^ software 1.5 followed by a visual control of accurate genotype classification.

### Statistics

Differences in categorical study variables were tested for statistical significance with the chi-squared test; for continuous variables *t*-tests and Kruskal–Wallis tests were applied. The Kolmogorov–Smirnov test was used as a test for normality. Non-normally distributed variables were log-transformed prior to analysis. Data are presented as means ± SD of not log-transformed values. Association between analyzed SLC5A2 SNPs with T2DM or CAD was determined by univariable and multivariable logistic regression analysis. Hazard ratios (HR) for the incidence of vascular events were derived from Cox proportional hazards models. Multiple linear regression analysis was used for assessing the association between genetic variants and continuous variables.

*P*-values < 0.05 were considered as significant. Statistical analyses were performed with the software package IBM SPSS 22 for Windows (IBM Corp., Armonk, NY, U.S.A.). Hardy–Weinberg equilibrium was assessed using chi-squared test with one degree of freedom.

### Meta-analysis

We performed a systematic literature search in the PubMed and the Cochrane libraries, including all published literature up to December 4th, 2018. The following search terms were used: Diabetes AND (SLC5A2 OR SGLT2) AND (‘single nucleotide polymorphism’ OR ‘genetic variation’). Records providing genotype frequencies of SLC5A2 SNPs for patients with T2DM and without T2DM were considered. The quality of enrolled studies was independently assessed by two investigators according to the Newcastle-Ottawa Scale (NOS) for quality assessment of case–control studies [25]. The maximum possible score is nine stars and studies with stars equal to or higher than five were considered to be of sufficient quality.

Odds ratios (ORs) together with their corresponding 95% confidence intervals (CIs) were adopted from previous reports or calculated based on reported genotype frequencies by logistic regression analysis for an additive model of inheritance. Meta-analysis was performed with the Comprehensive Meta-Analysis 3.3 statistical software (Biostat, Inc., Englewood, NJ, U.S.A.). Heterogeneity among studies was evaluated with the Cochran *Q* test and Higgins I2 statistic [26]. Fixed effects models were used in cases of low inter-study heterogeneity (*I2* < 50%, *P-*value > 0.05) to estimate the pooled additive ORs and 95% CIs. Otherwise, random effects models were applied. For publication bias assessing, rank correlation test by Begg and Mazumdar as well as Egger’s regression test were applied.

## Results

### Patient characteristics

Clinical and biochemical characteristics of the total study population, of patients with and of subjects without T2DM, and of patients with and of subjects without significant CAD are given in Table 1. Overall, the characteristics of our patients were representative for patients undergoing coronary angiography for the evaluation of CAD, with a high proportion of male gender and a high prevalence of a history of smoking, MetS, hypertension, and T2DM. Age, BMI, MetS, any lesions of the coronary arteries, significant coronary artery stenoses, total cholesterol and triglyceride levels as well as use of antihypertensive therapy were significantly associated with the presence of T2DM. Age, male gender, MetS, T2DM, total cholesterol, triglyceride levels as well as use of statins, anticoagulant therapy orantihypertensive therapy were significantly associated with the presence of significant CAD. Genotyping of SLC5A2 SNPs was successful in all patients and all analyzed variants were in Hardy–Weinberg equilibrium. Individual genotypes together with clinical data are given in Supplementary Table S1.

**Table 1:** Clinical and biochemical characteristics of the total study and of patients with and without type 2 diabetes mellitus

	All patients (*n* = 1684)	Non-diabetic patients (*n* = 1284)	Patients with T2DM (*n* = 400)	*P*-value	Patients without significant CAD (*n* = 710)	Patients with significant CAD (*n* = 974)	*P*-value
Age (years)	63.7	63.3	65.1	0.001	62.5 ± 10.6	64.6 ± 10.6	<0.001
Male gender (%)	66.7	66.1	68.5	0.378	52.4	77.1	<0.001
BMI (kg/m^2^)	27.6 ± 4.3	27.1 ± 4.1	29.1 ± 4.8	<0.001	27.9 ± 4.6	27.3 ± 4.1	0.007
Metabolic syndrome (%)	44.2	36.6	68.5	<0.001	40.8	46.6	0.019
Hypertension (%)	82.6	81.6	85.9	0.050	80.8	83.9	0.103
Type 2 diabetes (%)	23.8	–	–	–	18.5	27.6	<0.001
History of smoking (%)	59.2	57.5	66.0	0.002	51.3	65.5	<0.001
Any lesion of the coronary arteries (%)	81.7	79.6	88.3	<0.001	56.5	100.0	<0.001
Significant stenosis (%)	57.8	54.9	67.3	<0.001	–	–	–
Cholesterol (mg/dl)	204.0 ± 46.2	207.9 ± 45.2	191.3 ± 47.4	<0.001	208.0 ± 44.5	200.1 ± 47.3	0.002
Triglycerides (mg/dl)	149.0 ± 96.9	141.9 ± 89.9	171.6 ± 113.7	<0.001	142.6 ± 96.4	153.7 ± 97.0	0.003
eGFR (ml/min/1.73 m^2^)	93.4 ± 19.1	93.7 ± 18.8	92.2 ± 20.1	0.134	94.1 ± 18.1	92.8 ± 19.8	0.107
Statin therapy (%)	32.5	30.5	39.9	0.034	35.1	54.2	<0.001
ASA therapy (%)	68.5	57.5	71.9	0.104	66.1	70.3	0.074
Anticoagulant therapy (%)	7.5	6.8	9.7	0.057	10.2	5.5	<0.001
Antihypertensive therapy (%)	72.0	69.6	79.8	<0.001	66.6	76.0	<0.001

Data of patient characteristics are given as means ± standard deviations or percentage as indicated. Coronary angiography was performed with the Judkin’s technique and stenoses narrowing ≥ 50% were defined as significant CAD. Differences in categorical study variables between non-diabetic patients and patients with T2DM were tested for statistical significance with the chi-squared test; for continuous variables, *t*-tests were applied. Non-normally distributed variables (age, BMI, triglycerides, eGFR) were log-transformed prior to statistical analysis.

Abbreviations: ASA, acetylsalicylic acid; BMI, body mass index; CAD, coronary artery disease; eGFR, estimated glomerular filtration rate; T2DM, type 2 diabetes mellitus.

### Association of SLC5A2 variants with parameters of glucose homeostasis

The association between SLC5A2 variants and parameters of glucose homeostasis is shown in Table 2. Variant rs9934336 was significantly associated with decreased HbA1c, fasting glucose, and 120 min glucose concentrations during oral glucose tolerance tests. Variant rs3813008 was significantly linked to 120 min glucose during oral glucose tolerance tests. No significant associations between variant rs3116150 and the investigated traits of glucose homeostasis were observed.

**Table 2:** Association between analyzed SLC5A2 single nucleotide polymorphisms and traits of glucose hemostasis in the total study cohort

	*n*	HbA1c (%)	Fasting glucose (mmol/l)	120 min glucose (mmol/l)	Fasting insulin (µU/ml)	HOMA IR	HOMA BCF
rs9934336							
GG	939 (55.8%)	6.13 ± 1.05	6.3 ± 2.06	8.04 ± 4.37	13.14 ± 30.92	4.13 ± 14.77	104.42 ± 117.4
GA	638 (37.9%)	6.06 ± 0.96	6.16 ± 1.88	7.7 ± 4.11	11.47 ± 10.61	3.44 ± 4.91	98.98 ± 71.26
AA	107 (6.4%)	5.94 ± 0.78	5.93 ± 1.29	7.11 ± 3.19	10.98 ± 8.13	3.12 ± 3.11	96.49 ± 52.93
*P*-value		0.023	0.044	0.049	0.235	0.075	0.584
rs3813008							
GG	1267 (75.2%)	6.09 ± 1.01	6.2 ± 1.9	7.66 ± 4.03	12.56 ± 26.91	3.92 ± 12.94	102.73 ± 103.75
GA	395 (23.5%)	6.09 ± 0.95	6.28 ± 2.07	8.35 ± 4.62	11.64 ± 10.03	3.36 ± 3.18	99.42 ± 81.47
AA	22 (1.3%)	6.36 ± 1.46	6.71 ± 2.91	9.47 ± 5.89	12.59 ± 9.76	4.62 ± 6.28	90.42 ± 54.84
*P*-value		0.355	0.221	0.019	0.626	0.880	0.329
rs3116150							
GG	940 (55.8%)	6.06 ± 0.99	6.18 ± 1.96	7.92 ± 4.23	11.51 ± 9.68	3.41 ± 4.15	99.81 ± 80.95
GA	634 (37.6%)	6.12 ± 1.01	6.27 ± 1.96	7.69 ± 4.17	13.76 ± 37.3	4.44 ± 17.94	105.56 ± 124.23
AA	110 (6.5%)	6.18 ± 1.05	6.28 ± 1.84	8.05 ± 4.25	11.66 ± 8.12	3.51 ± 3.42	97.82 ± 68.3
*P*-value		0.147	0.239	0.513	0.377	0.255	0.898

Analyses were performed by multiple linear regression analysis using sex, age, and body mass index as covariates under an additive model of inheritance. All variables were log-transformed prior to statistical analysis. Data are presented as mean ± SD of non-log-transformed values.

Abbreviations: HbA1c, hemoglobin A1c; HOMA BCF, homeostasis model of assessment beta-cell function; HOMA IR, homeostasis model assessment of insulin resistance; SD, standard deviation.

### Association of SLC5A2 variants with type 2 diabetes

**Figure 1:**
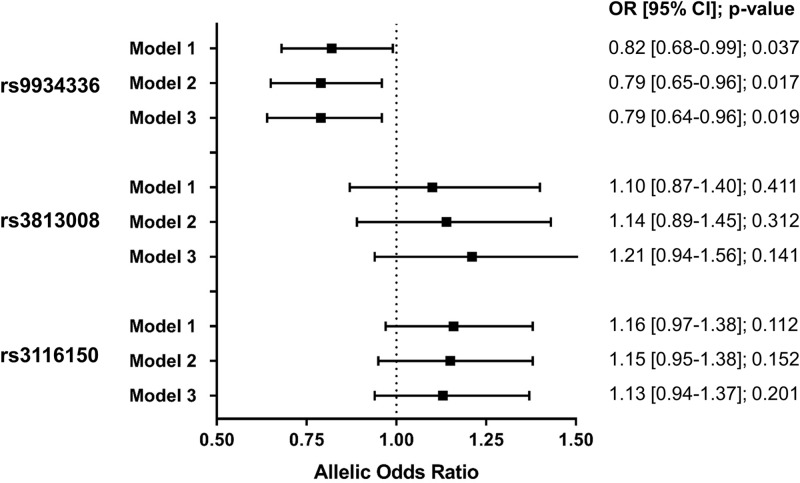
Association between analyzed SLC5A2 variants and type 2 diabetes mellitus determined by logistic regression analysis Model 1, unadjusted; model 2, adjusted for sex, age, and body mass index; model 3, additionally adjusted for metabolic syndrome and hypertension. CI, confidence interval; OR, odds ratio.

The association between the analyzed SLC5A2 variants and T2DM is displayed in Figure 1. Variant rs9934336 was significantly associated with T2DM univariately as well as in multivariable logistic regression analyses adjusting for sex, age, BMI, the metabolic syndrome, and hypertension. Variants rs3813008 and rs3116150 did not show any significant correlation with T2DM.

### Results from meta-analysis

A systematic review of the literature identified four original articles [10,11,27,28] and one review article [29] for the given search terms. Two original articles [10,11] provided genotype frequencies of SLC5A2 SNPs for patients with and subjects without T2DM and were scheduled for the meta-analysis (Supplementary Figure S1).

The study by Enigk et al. [11] included two study cohorts comprising 786 non-diabetic Sorbs (a self-contained population in Eastern Germany) and 106 Sorbs with T2DM in the first and 1683 non-diabetic subjects and 359 T2DM patients from Berlin in the second cohort.

However, only for the first cohort genotyping data in conjunction with the presence or absence of T2DM were reported. Therefore, the second cohort was not considered in the meta-analysis. The study by Zimdahl et al. [10] included 2229 individuals without diabetes, but at increased risk for type 2 diabetes (defined as a family history of type 2 diabetes, BMI of at least 27 kg/m^2^, impaired fasting glycemia, and/or previous gestational diabetes) and 979 patients with T2DM included in placebo-controlled phase III clinical trials, which assessed the safety and efficacy of the SGLT2-inhibitor empagliflozin.

According to NOS criteria both studies were of sufficient quality (eight and six stars for the study by Enigk et al. [11] and Zimdahl et al. [10], respectively) and were, therefore, included in our meta-analysis. Consequently, meta-analysis of these two previous studies together with the present study included a total of 5196 non-diabetic subjects and 1738 individuals with T2DM. Genotype distributions among studies included in meta-analysis are given in Supplementary Table S1.

Heterogeneity assessment showed no significant inter-study heterogeneity for variants rs9934336 and rs3813008 (I2 = 0.0%; *P* = 0.510 and I2 = 0.0%; *P* = 0.682, respectively); therefore fixed effects models were used to estimate the pooled additive ORs and 95% CIs. A significant heterogeneity between studies was observed for variant rs3116150 (I2 = 91.2%; *P* ≤ 0.001), so a random effects model was used. Begg and Mazumdar’s rank correlation test as well as Egger’s regression test indicated that there were no publication bias in any performed meta-analysis (all *P*-values >0.05; Supplementary Table S2).

**Figure 2:**
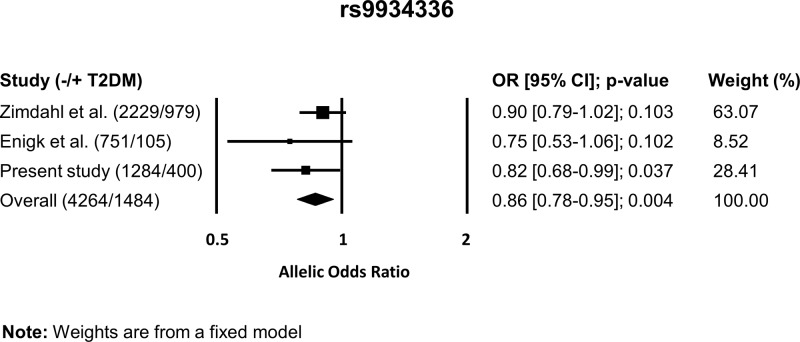
Meta-analysis of the association between rs9934336 and type 2 diabetes mellitus Weights are from a fixed model. Abbreviations: CI, confidence interval; OR, odds ratio.

ORs of the individual studies as well as the pooled OR are shown for rs9934336 in Figure 2. The association between rs9934336 and T2DM did not reach statistical significance in the two previously performed studies. However, in the meta-analysis the pooled OR of rs9934336 of the previously published observations together with the data from our study was highly significantly associated with T2DM, reaching an approximately 10-fold increase in the level of statistical significance as compared with the data from our study alone. Notably, the association between rs9934336 and T2DM in the non-definite previously published single observations remained significant after exclusion of the present study from the meta-analysis (OR = 0.88 [0.78–0.99]; *P* = 0.036. Also after individual removal of the other studies the pooled OR remained significant (OR = 0.80 [0.68–0.95]; *P* = 0.009 and 0.87 [0.79–0.97]; *P* = 0.012 by removing the study by Zimdahl et al. and Enigk et al., respectively).

No significant association of variant rs3813008 and rs3116150, respectively, with T2DM was observed in the meta-analysis (OR = 1.01 [0.89–1.14]; *P* = 0.870 and OR = 0.92 [0.63–1.34]; *P* = 0.667, respectively; Figures S3 and S4). It should be noted, however, that in the study by Zimdahl et al. the association between rs3116150 and T2DM was significant, but contrary to the (non-significant) effect of the other studies, probably leading to the observed inter-study heterogeneity among studies.

### Associations of SLC5A2 variants with coronary atherosclerosis and with the incidence of cardiovascular events

None of the tested SLC5A2 genetic variants was significantly associated with the presence of significant CAD at baseline, either in univariate (Table 3) or in multivariable logistic regression analysis (Supplementary Table S3). To further evaluate their quantitative contribution to angiographically characterized coronary atherosclerosis, we investigated the association of these genetic variants with the extent of coronary atherosclerosis. No significant association of SLC5A2 SNPs with the extent of coronary atherosclerosis were found (Supplementary Table S4).

**Table 3:** Association of SLC5A2 variants with significant coronary artery disease

SNP	Genotype frequencies (controls/cases)			OR [95% CI]	*P*-value
	AA	AB	BB		
rs9934336	396/543	264/374	50/57	0.97 [0.83–1.14]	0.701
rs3813008	532/735	167/228	11/11	0.96 [0.78–1.18]	0.680
rs3116150	397/543	263/371	50/60	0.98 [0.84–1.15]	0.814

Association between analyzed SLC5A2 variants and significant coronary stenosis determined by univariable logistic regression analysis under an additive model of inheritance.

Abbreviations: A, major allele, B, minor allele; CI, confidence interval; OR, odds ratio; SNP, single nucleotide polymorphism.

Prospectively, a follow-up rate of 98.6% was achieved. From 1684 patients investigated at baseline, 1661 therefore entered the prospective analyses. Prospectively, cardiovascular events occurred in 30.1% of our patients during the follow-up period of 7.6 ± 2.7 years. None of the tested genetic variants was significantly associated with the incidence of future cardiovascular events, either in univariate (Table 4) or in multivariable Cox regression analysis (Supplementary Table S5).

**Table 4:** Association of SLC5A2 variants with the incidence of future cardiovascular events

SNP	Genotype frequencies (controls/cases)			HR [95% CI]	*P*-value
	AA	AB	BB		
rs9934336	624/300	442/189	73/33	0.94 [0.82-1.09]	0.410
rs3813008	855/394	269/121	15/7	0.96 [0.80-1.15]	0.650
rs3116150	637/288	432/195	70/39	1.05 [0.92-1.21]	0.478

Association between analyzed SLC5A2 variants and the incidence of future cardiovascular events was determined by univariable Cox regression analysis under an additive model of inheritance. Abbreviations: A, major allele, B, minor allele; CI, confidence interval; HR, hazard ratio; SNP, single nucleotide polymorphism.

To investigate the interaction effect of SLC5A2 SNPs and diabetes on the cardiovascular risk we conducted tests for interaction and performed subgroup analyses with respect to the presence or absence of T2DM. Neither in non-diabetic subjects nor in patients with T2DM SLC5A2 SNPs were significantly associated with the prevalence of significant coronary atherosclerosis (Supplementary Table S6) or the incidence of future vascular events (Supplementary Table S7). Thus, no significant interaction terms between SLC5A2 SNPs and diabetes regarding the cardiovascular risk were observed (all *P*_interaction_ values ≥ 0.05).

## Discussion

The present work is novel in several aspects. First, we for the first time observed a significant association of SLC5A2 SNPs with T2DM. Second, together with previously published data from the literature, which by themselves did not reveal this association we performed a meta-analysis which confirmed our novel finding. Third, this is the first investigation prospectively addressing the association of SLC5A2 SNPs with cardiovascular events. We found a significant association between the common genetic variant rs9934336 of the SGLT2-encoding gene SLC5A2 and fasting glucose, post-challenge glucose, HbA1c and with the presence of T2DM in angiographied coronary patients. In contrast, no significant association between this SNP and CAD or with the incidence of cardiovascular events could be observed over 7.6 ± 2.7 years of follow-up.

Results from single investigations on SNPs are now rightfully interpreted with caution because they previously often could not be reproduced in other cohorts. In this regard, other single studies failed to detect the association between rs9934336 and T2DM. Several reasons may have contributed to the different strength of association among studies: First, differences in patients’ characteristics (including e.g. age, gender distribution, etc.) between studies may have confounded the association between SLC5A2 SNPs and T2DM. Furthermore, our study population consists of angiographied coronary patients and results obtained from this high coronary risk cohort are not necessarily applicable to other populations. On the other side, the study by Zimdahl et al. [10] included subjects without diabetes, but at increased risk for type 2 diabetes. Therefore, genetic differences between non-diabetic subjects at high diabetic risk and patients with diabetes may be attenuated in the present study. Subjects investigated by Enigk et al. [11] are part of a self-contained population, the Sorbs. Here, it can be speculated that the genetic background of diabetes differs between ethnicities.

However, our meta-analysis including previously published data concerning SLC5A2 variants and T2DM [10,11] supports our findings demonstrating a significant association between rs9934336 and T2DM. Notably, in this meta-analysis rs9934336 was associated with the presence of T2DM both with and without inclusion of our data.

The observations we made can be attributed to a distinct pathophysiological mechanism. Like in familial renal glucosuria, lowered function of SGLT2 leads to glucose spillage over the urine and prevents larger glucose increases, particularly in the postprandial state. In the long-term, this mechanism should be expected to protect from the development of T2DM. This idea is supported by the benign natural history of familial renal glucosuria [2] and of renal glucosuria in pregnancy [30].

The magnitude of glucose excretion is increased up to 74% of the filtered load [1] in diabetic patients administered empagliflozin but has not been investigated for the genetic variant rs9934336. Glucose excretion can be expected to be much less pronounced with the genetic variant than with the pharmacological intervention. This could explain why in contrast with the EMPAREG-OUTCOME trial, where empagliflozin led to a significant reduction in cardiovascular events and total mortality, no significant reduction in cardiovascular events was noted in our investigation, neither in the total patient cohort nor in in subgroup analyses stratified by the diabetic state, although we, similar to EMPAREG-OUTCOME, investigated a high-risk patient population. Here it should be noted, that sample size, particularly in subgroup analysis, appears small and, therefore, from our data we cannot exclude that an association between rs9934336 and cardiovascular events could emerge in a much larger study. Of note, however, no data on the association of the polymorphism with CAD or with cardiovascular events are available from the literature. In face of the results of outcome studies showing cardiovascular risk reductions with SGLT2 inhibitors, it would be of particular interest to further investigate the association between genetic variation at the SLC5A2 gene locus and cardiovascular disease in larger study cohorts in order to definitely clarify this issue.

However, it should be considered that also in trials investigating cardiovascular outcomes with SGLT2 inhibitors [4–6], these medications improved heart failure outcomes and cardiovascular mortality but not typical atherothrombotic endpoints such as myocardial infarctions or strokes. Most likely the beneficial impact of SGLT2 inhibitors on cardiovascular outcomes is not carried by an anti-atherosclerotic effect which is very well in line with the lack of an association between SLC5A2 SNPs and CAD in our study.

We observed a significant association between rs9934336 and T2DM. However, it may be questioned whether the assumed moderate impact of common genetic variants on glucose levels via its effect on SGLT2 function is sufficient to protect from diabetes. In this context it must not be forgotten that a genetic variant exerts its effect from birth on over a lifetime and that even slight effects can have a huge impact on long term prognosis. Here, a comparison with loss-of-function mutations in other metabolic fields appears tempting. Noteworthy, it has been shown that common variants in the genes encoding for Niemann–Pick C1-like 1 and Proprotein Convertase Subtilisin Kexin 9, critical proteins in cholesterol metabolism only slightly decrease LDL cholesterol but have a huge impact on the lifelong incidence of cardiovascular events [31–33]. Similarly, it appears plausible that live-long exposure to slightly reduced plasma glucose concentrations and to a slightly increased loss of calories in the urine caused by genetic variants lowering SGLT2 activity may protect from the development of T2DM.

An in-depth molecular genetic consideration appears necessary. Variant rs9934336 is located in intron 1, 121 base-pairs upstream of the 5′ end of exon 2. *In silico* splice site analysis using Human Splicing Finder 3.0 tool (www.umd.be/HSF3) [34] showed that the variant creates a new intronic splicing enhancer site, but probably has no impact on splicing due to its deep intronic position. Therefore, a potential impact of rs9934336 on SGLT2 protein structure remains unclear and it appears more likely that a variant highly correlated with it may contribute to putatively decreased SGLT2 activity.

Our study has strengths and limitations. Important strengths of this work are a broad characterization of glucose metabolism including besides fasting glucose HbA1c, in oral glucose tolerance tests, and measures of insulin resistance and beta-cell function. Further, the population of angiographied coronary patients we chose to investigate allowed us to study the association of polymorphisms both with coronary atherosclerosis and with the incidence of cardiovascular events. Further, our high-risk patient population has been of particular interest in randomized clinical trials using SGLT2 inhibitors [4,6]. Furthermore, we have not accounted for multiple testing in our study. Of note, the Bonferroni correction would probably have been too conservative owing to the given close correlation among included SLC5A2 SNPs and investigated phenotypes (T2DM, coronary atherosclerosis, cardiovascular events). Therefore, associations between analyzed SNPs and clinical outcome are given for a nominal significance level. Notably, the observed significant association between variant rs9934336 and T2DM obtained by meta-analysis would have survived Bonferroni correction (*P*_corrected_ = 0.036). As stated above, we cannot exclude that some associations not found to be significant in our study may reach statistical significance with a larger sample size. Clearly, replication of our observations in large independent studies is warranted. It should be further mentioned that the observed associations between determined genotypes and glycemic traits might have been influenced by the prevalence of T2DM or its treatment. Furthermore, like in other studies on common SLC5A2 variants [10,11,28], data on the extent of urinary glucose excretion are not available in our study, which however of course does not diminish the validity of our results. From our data we also cannot exclude that SLC5A2 polymorphisms are associated with microvascular complications. SLC5A2 SNPs were associated with the presence of T2DM in our patients as well as in our meta-analysis. Whether SLC5A2 SNPs in a pure diabetes cohort are associated with microvascular diabetes complications is an important question that should be addressed in large cohorts of patients with diabetes.

## Conclusions

In conclusion, thus, we have presented novel data pointing to a significant role of a SLC5A2 genetic variant in glucose homeostasis. The variant rs9934336 is associated with decreased presence of diabetes in cardiac patients. Our finding is supported by a meta-analysis including previously published data. Hitherto neglected, the SGLT2 transport mechanism should gain in-depth research interest with larger studies of genomics, downstream effects and functional consequences of the protective SNP described here. A new field of clinical research for diabetologists, nephrologists, and cardiologists emerges.

## Supporting information

**Supplementary Figure S1:** Flow chart of the literature selection process in the meta-analysis

**Supplementary Table S1:** Genotype distributions among studies included in meta-analyses

**Supplementary Table S2:** Evaluation of publication bias

**Supplementary Table S3:** Association between SLC5A2 SNPs and significant coronary atherosclerosis - results from multivariable logistic regression analyses

**Supplementary Table S4:** Associations between SLC5A2 SNPs and the extend of significant coronary lesions

**Supplementary Table S5:** Association between SLC5A2 SNPs and significant coronary atherosclerosis - results from multivariable Cox regression analyses

**Supplementary Table S6:** Association of SLC5A2 variants with significant coronary artery disease in non-diabetic patients and patients with type 2 diabetes

**Supplementary Table S7:** Association of SLC5A2 variants with future cardiovascular events in non-diabetic patients and patients with type 2 diabetes

## Abbreviations

BMI: body mass index
CAD: coronary artery disease
CI: confidence interval
EMPA-REG-OUTCOME: Empagliflozin, Cardiovascular Outcomes, and Mortality in Type 2 Diabetes
NOS: Newcastle-Ottawa Scale
OR: odds ratio
SGLT2: sodium glucose co-transporter 2
SNP: single nucleotide polymorphism
T2DM: type 2 diabetes mellitus
