# Supplementary material for: Are SGLT2 polymorphisms linked to diabetes mellitus and cardiovascular disease? Prospective study and meta-analysis
Source: Biosci Rep. 2019 Aug 7;39(8):BSR20190299. doi: 10.1042/BSR20190299 (PMC6684948; doi:10.1042/BSR20190299)
Supplement: Supplementary file 1 [file bsr20190299_Supp1.pdf]

S1 Fig: Flow chart of the literature selection process in the meta-analysis

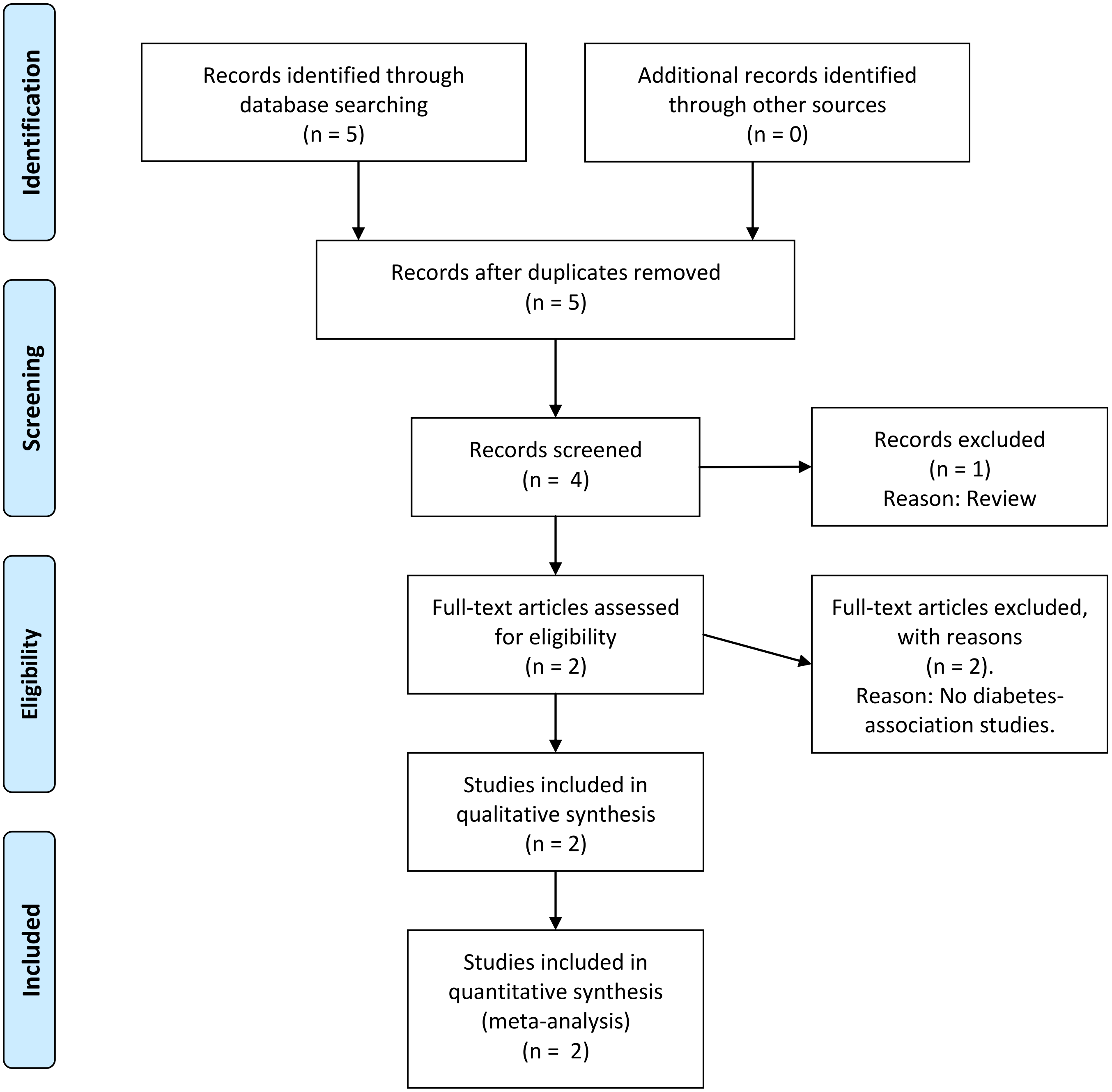

rs3813008

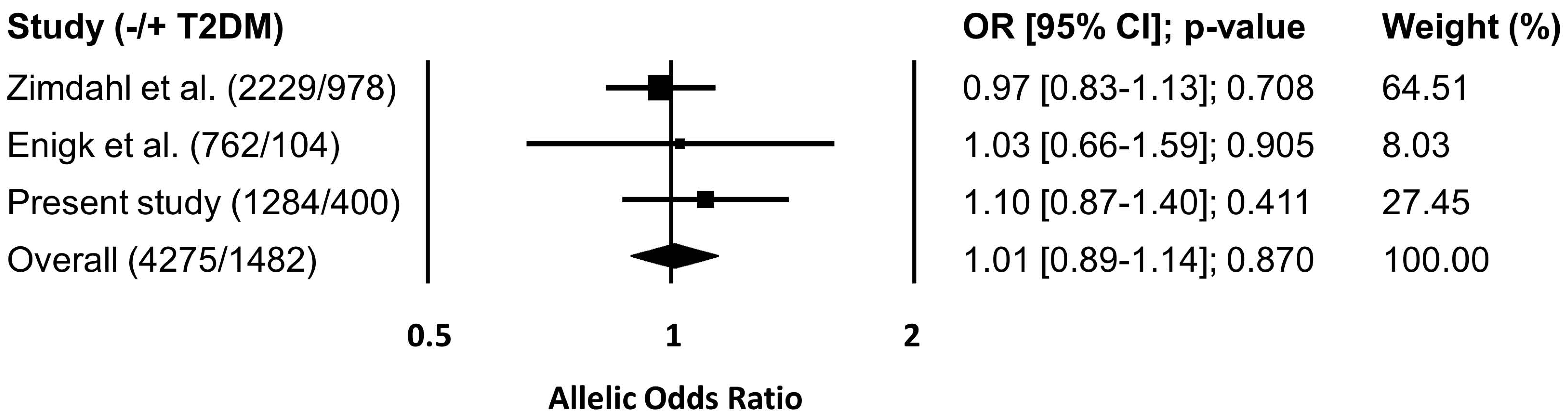

Note: Weights are from a fixed model

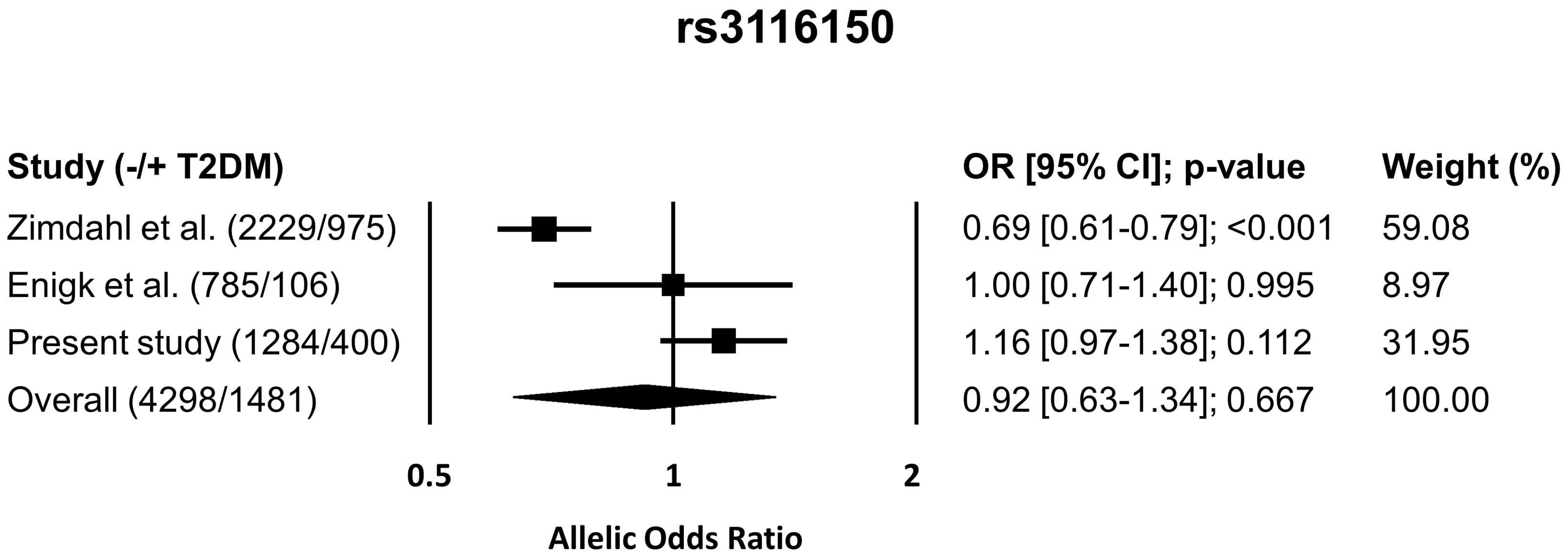

Note: Weights are from a random-effect model

S1 Table: Genotype distributions among studies included in meta-analyses

Study	rs9934336 (controls/cases)			HWE p-value
	GG	GA	AA	
Enigk et al.	390/65	305/33	56/7	1.00
Zimdahl et al.	1251/572	843/362	135/45	0.299
Present study	699/240	498/140	87/20	0.920
	rs3813008 (controls/cases)			
	GG	GA	AA	
Enigk et al.	588/80	162/22	12/2	0.740
Zimdahl et al.	1643/727	543/233	43/18	0.791
Present study	972/295	296/99	16/6	0.155
	rs3116150 (controls/cases)			
	GG	GA	AA	
Enigk et al.	457/64	286/34	42/8	0.920
Zimdahl et al.	1204/625	881/320	144/35	0.320
Present study	730/210	474/160	80/30	0.823

HWE, Hardy-Weinberg equilibrium

S2 Table: Evaluation of publication bias

	Begg-Mazumdar rank correlation test, p-value	Egger's regression test, p-value
rs9934336	0.117	0.200
rs3813008	0.602	0.643
rs3116150	0.602	0.615

P-values were two-tailed. The Begg and Mazumdar rank correlation test was applied without continuity correction.

S3 Table: Association between SLC5A2 SNPs and significant coronary atherosclerosis - results from multivariable logistic regression analyses

SNP	Adjustment model	OR [95% CI]	p-value
rs9934336	1	0.98 [0.83-1.15]	0.773
	2	0.99 [0.84-1.18]	0.930
rs3813008	1	0.92 [0.74-1.14]	0.448
	2	0.89 [0.71-1.11]	0.287
rs3116150	1	0.99 [0.84-1.17]	0.908
	2	0.96 [0.81-1.15]	0.679

Adjustment model 1 adjusts for age, sex, and body mass index; model 2 adjusts for age, sex, body mass index, type 2 diabetes mellitus, hypertension, smoking, LDL cholesterol, and HDL cholesterol. OR, odds ratio; CI, confidence interval.

S4 Table: Associations between SLC5A2 SNPs and the extend of significant coronary lesions

SNP	Genotype	Mean (±SD)	Number of significant stenoses										p-value
			0	1	2	3	4	5	6	7	8	9	
rs599839	GG	1.37 ± 1.57	396	176	154	126	47	19	12	7	1	1	
	GA	1.44 ± 1.69	263	127	103	64	45	19	7	5	4	1	0.423
	AA	1.23 ± 1.62	50	19	19	9	5	2	0	1	0	1	
rs3813008	GG	1.40 ± 1.61	531	232	217	155	71	32	12	10	4	2	
	GA	1.38 ± 1.66	167	86	54	42	26	8	7	3	1	1	0.474
	AA	0.91 ± 1.06	11	4	5	2	0	0	0	0	0	0	
rs3116150	GG	1.37 ± 1.62	396	187	154	101	57	21	11	7	3	2	
	GA	1.40 ± 1.60	263	117	105	86	34	17	4	5	2	1	0.810
	AA	1.39 ± 1.71	50	18	17	12	6	2	4	1	0	0	

Analyses were performed by Kruskal-Wallis tests under an additive model of inheritance. The extent of significant CAD was defined as the number of significant coronary stenoses (lumen narrowing ≥50%) in a given patient.

S5 Table: Association between SLC5A2 SNPs and significant coronary atherosclerosis - results from multivariable Cox regression analyses

SNP	Adjustment model	HR [95% CI]	p-value
rs9934336	1	0.93 [0.81-1.07]	0.311
	2	0.94 [0.82-1.09]	0.438
rs3813008	1	0.95 [0.79-1.14]	0.585
	2	0.93 [0.77-1.12]	0.430
rs3116150	1	1.05 [0.92-1.21]	0.476
	2	1.06 [0.92-1.22]	0.416

Adjustment model 1 adjusts for age, sex, and body mass index; model 2 adjusts for age, sex, body mass index, type 2 diabetes mellitus, hypertension, smoking, LDL cholesterol, and HDL cholesterol. HR, hazard ratio; CI, confidence interval.

S6 Table: Association of SLC5A2 variants with significant coronary artery disease in non-diabetic patients and patients with type 2 diabetes

SNP	T2DM	Genotype frequencies (controls/cases)			OR [95% CI]	p-value	$p_{interaction}$-value
		AA	AB	BB			
rs9934336	No	318/381	217/281	44/43	0.98 [0.82-1.18]	0.859	0.943
	Yes	78/162	47/93	6/14	1.00 [0.70-1.42]	0.993	
rs3813008	No	435/537	136/160	8/8	0.94 [0.74-1.19]	0.859	0.912
	Yes	97/198	31/68	3/3	0.97 [0.63-1.49]	0.886	
rs3116150	No	332/398	211/263	36/44	1.03 [0.86-1.23]	0.791	0.193
	Yes	65/145	52/108	14/16	0.80 [0.58-1.11]	0.180	

Associations between analysed SLC5A2 variants and significant coronary stenosis were determined by logistic regression analysis under an additive model of inheritance. SNP, single nucleotide polymorphism; A, major allele, B, minor allele; OR, odds ratio, CI, confidence interval.

S7 Table: Association of SLC5A2 variants with future cardiovascular events in non-diabetic patients and patients with type 2 diabetes

SNP	T2DM	Genotype frequencies (controls/cases)			HR [95% CI]	p-value	$p_{interaction}$-value
		AA	AB	BB			
rs9934336	No	483/205	352/141	62/24	0.96 [0.81-1.13]	0.581	0.942
	Yes	141/95	90/48	11/9	0.97 [0.73-1.28]	0.809	
rs3813008	No	681/276	206/88	10/6	1.04 [0.84-1.29]	0.689	0.139
	Yes	174/118	63/33	5/1	0.75 [0.53-1.07]	0.117	
rs3116150	No	508/212	338/130	51/28	1.02 [0.87-1.21]	0.807	0.661
	Yes	129/76	94/65	19/11	1.09 [0.85-1.40]	0.479	

Associations between analysed SLC5A2 variants and significant coronary stenosis were determined by Cox regression analysis under an additive model of inheritance. SNP, single nucleotide polymorphism; A, major allele, B, minor allele; HR, hazard ratio, CI, confidence interval.
